# Segmentation of the thoracolumbar fascia in ultrasound imaging: a deep learning approach

**DOI:** 10.1186/s12880-025-01720-2

**Published:** 2025-05-15

**Authors:** Lorenza Bonaldi, Carmelo Pirri, Federico Giordani, Chiara Giulia Fontanella, Carla Stecco, Francesca Uccheddu

**Affiliations:** 1 Department of Civil, Environmental and Architectural Engineering, University of Padova, 35131 Padova, Italy; 2 Center for Mechanics of Biological Materials, University of Padova, 35131 Padova, Italy; 3 Department of Neuroscience, Institute of Anatomy, University of Padova, 35131 Padova, Italy; 4 Neurological Rehabilitation Centre, Villa Rosa, Trento, Italy; 5 Department Industrial Engineering, Center for Mechanics of Biological Materials, University of Padova, 35131 Padova, Italy

**Keywords:** Segmentation, Deep learning, Deep fascia, Thoracolumbar, Low back pain

## Abstract

**Background:**

Only in recent years it has been demonstrated that the thoracolumbar fascia is involved in low back pain (LBP), thus highlighting its implications for treatments. Furthermore, an easily accessible and non-invasive way to investigate the fascia in real time is the ultrasound examination, which to be reliable as is, it must overcome the challenges related to the configuration of the machine and the experience of the operator. Therefore, the lack of a clear understanding of the fascial system combined with the penalty related to the setting of the ultrasound acquisition has generated a gap that makes its effective evaluation difficult during clinical routine. The aim of the present work is to fill this gap by investigating the effectiveness of using a deep learning approach to segment the thoracolumbar fascia from ultrasound imaging.

**Methods:**

A total of 538 ultrasound images of the thoracolumbar fascia of LBP subjects were finally used to train and test a deep learning network. An additional test set (so-called Test set 2) was collected from another center, operator, machine manufacturer, patient cohort, and protocol to improve the generalizability of the study.

**Results:**

A U-Net-based architecture was demonstrated to be able to segment these structures with a final training accuracy of 0.99 and a validation accuracy of 0.91. The accuracy of the prediction computed on a test set (87 images not included in the training set) reached the 0.94, with a mean intersection over union index of 0.82 and a Dice-score of 0.76. These latter metrics were outperformed by those in Test set 2. The validity of the predictions was also verified and confirmed by two expert clinicians.

**Conclusions:**

Automatic identification of the thoracolumbar fascia has shown promising results to thoroughly investigate its alteration and target a personalized rehabilitation intervention based on each patient-specific scenario.

## Background

**Fig. 1:**
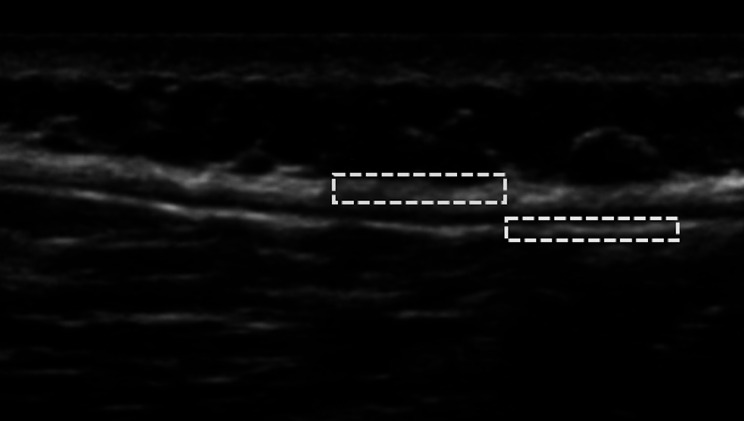
Thoracolumbar fascia of a low back pain subject, from US imaging. Selection: (blue) a portion of TLF composed by different sublayers, (yellow) a portion of epimysial fascia of the erector spinae muscles. The proper gliding between TLF and epimysium ensures forces transmission

Connective tissues (such as fascia) are hypothesized to play a key role in the pathogenesis of low back pain (LBP) [1–3]. In fact, the thoracolumbar fascia (TLF), i.e. a trilaminar structure that involves the back muscles, in subjects with LBP showed greater thickness and echogenicity compared to a control group of subjects without LBP [1, 4]. Furthermore, it has been shown that the shear stress of the thoracolumbar fascia, within its layers, is lower in human subjects with chronic LBP [5]. Despite the clinical importance of the fascial structure in disorders and consequently in treatment, it has long been considered a “forgotten structure” with difficulty for operators in its identification [6–8]. Consequently, studies quantitatively evaluating fascial tissues in this clinical condition were lacking. For the reasons mentioned above, an automatic segmentation of the fascial layers is essential to support their analysis. A rapid way to evaluate fascial structures in a static and dynamic scenario is ultrasound (see Fig. 1), but anatomical detection strictly relies on the experience and reliability of the operator [6]. Even more, an automatic segmentation of the fascial system in ultrasound imaging is a necessity.

Imaging segmentation offers a variety of methodological solutions in ultrasound domain [9, 10]. Taking advantage of the success of artificial intelligence, in recent years deep learning solutions have been implemented in various medical fields to solve the segmentation task, from computer-assisted surgery [11, 12], to treatment facilitation [13, 14]. Indeed, deep learning has proven to be a feasible paradigm for segmenting different musculoskeletal structures, even in ultrasound imaging [11–13, 15–20]. However, there is a lack of results in the literature regarding the segmentation of the fascial system, such as the thoracolumbar fascia of low back pain subjects, in this imaging modality.

The implication of the thoracolumbar fascia in the scenario of low back pain, the challenges related to its evaluation by ultrasound, the clinical need for automated solutions, the lack of results in the literature that combined these requirements, triggered the current study. Therefore, we considered this open topic (i.e., automated segmentation of the thoracolumbar fascia of subjects with low back pain using ultrasound) as the focus of current work that aims to offer a solution of practical utility for clinical applications and prospects.

## Methods

In this section, we present the dataset and network used to solve the task of TLF segmentation from ultrasound images.

### Dataset collection

This retrospective study was approved by the Universitätsklinikum Münster (ethics committee approval n° 2022-303-f-S) and informed consent was waived. All data were collected at the institute from June 2022 to December 2022 by the same operator who had more than 8 years of experience in the field of fascia evaluation and identification (F.G.). Patients eligible for inclusion were aged between 20 and 70 years with a diagnosis of chronic LBP (lasting more than 3 months with daily manifestations). 46 patients (28 females; 18 males) were enrolled in the study. The mean age of the study group was 58.50 years (y/o), ranging between a minimum of 25 years and a maximum of 70 years, with an average LBP duration of 60 months (ranging between a minimum of 3 months and a maximum of 540 months). The exclusion criteria were as follows: (1) neurological signs of disc herniation with sensorimotor impairment (2) severe spinal stenosis (3) structural lesions of grade 2 or higher spondylolisthesis (4) secondary vertebral lesions (5) neoplastic origin (6) vascular etiology. They underwent a single session of ultrasound examinations (machine manufacturer and model: Alpinion- ECUBE i7) of the TLF at the L3 level of the spinal erectors, bilaterally. The acquisition protocol [21] required the patient to be in the prone position (no movement). The spinous process of L2 was used as an anatomical landmark for the transverse acquisitions. Starting from this position, longitudinal acquisitions was captured after a 90° rotation on the central portion of the belly of the erector spinae. The acquired images/videos for each subject were at least one for each scan and body side. The patient group reflects a great variability regarding anthropometric parameters, pain level, disease duration and phenotype. Outliers were discarded from the total original image dataset by two operators with more than 8 years of experience in the field of fascia evaluation and identification (F.G. and C.P.). A total of 538 final images were used for the study. The latter images collection was then randomly split into training (360 images), validation (91 images) and testing (87 images) subsets (so-called *Test set 1)*. To improve network generalizability in different scenarios (a different center, operator, machine manufacturer, patient cohort, protocol), network performances were tested on an additional dataset (not included in the training set, so-called *Test set 2*) collected during 2023, by two expert clinicians in the field (C.P. and C.S.), according to the Helsinki Declaration and human experimentation rules [22], and the Ethics Committee of the University of Padua approved the research. Each volunteer was informed of the testing protocol and signed a written informed consent before starting the study. All data were collected anonymously. In this case, the exclusion criteria comprised a prior history of LBP and a pain that limits daily activities at lower limb, a history of spinal or lower extremity surgeries, spinal deformities, severe lower back pain, prior fractures in the spine or lower extremities, fibromyalgia, balance impairments, and systemic diseases such as rheumatological conditions or diabetes. The TLF acquisition protocol required the patient to perform a trunk extension and the ultrasound images were acquired at vertebral level L3 (transversal probe positioning orientation), randomly during the movement, 1 each patient. The manufacturer and model of the ultrasound machines was Edge II, Sonosite, FUJIFILM, Inc. 21,919, WA, USA, with a linear probe, 6-15 MHz. Finally, the included patients were 6 (1 male, 5 females), aged 18 to 30 years.

### Dataset preparation

**Fig. 2:**
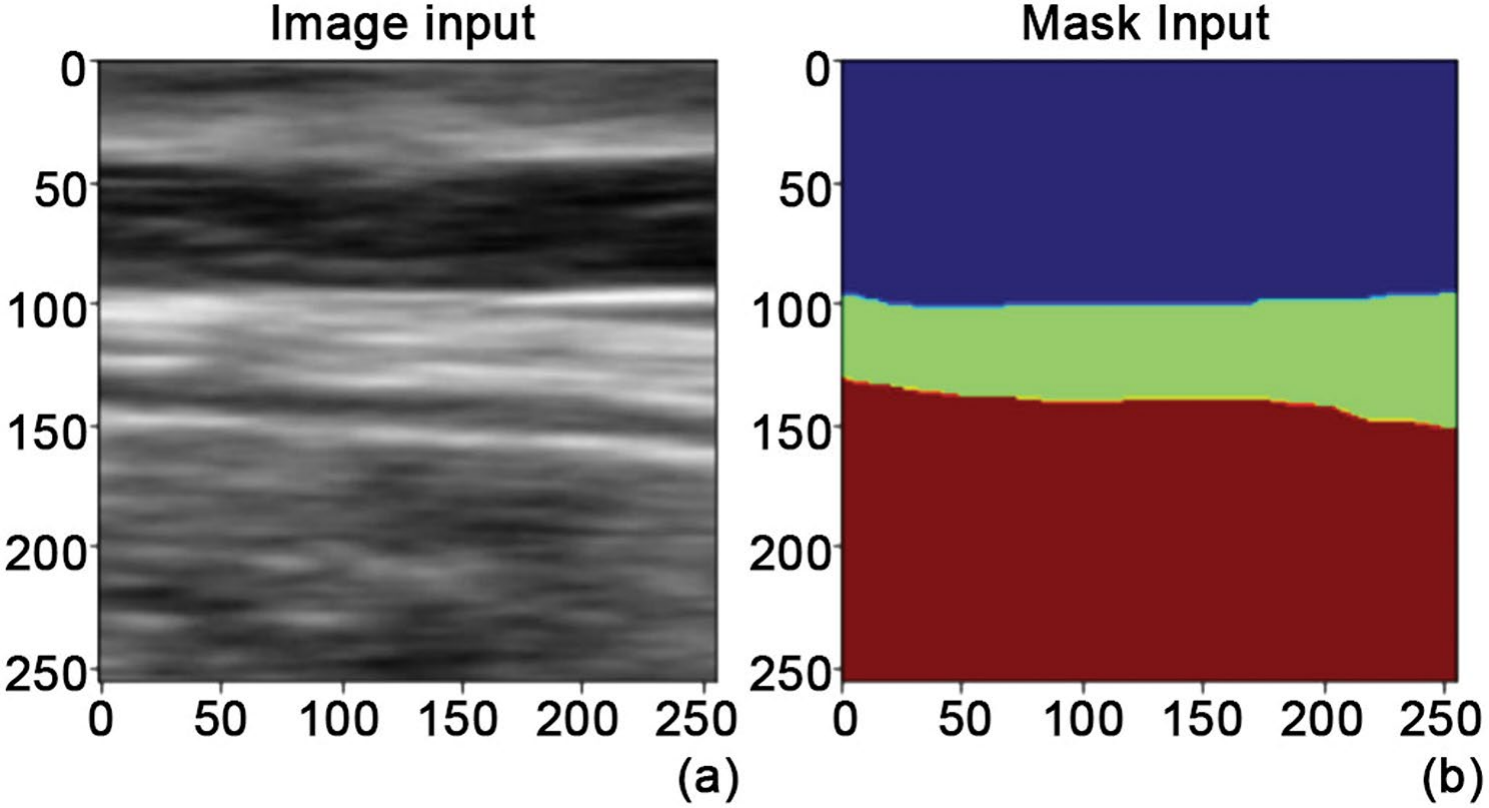
(**a**) Image and (**b**) mask (labelled) as inputs for the network

Each image of the final dataset collection was labelled (C.P.) in three classes with Apeer annotation tool [23]. The first class included the tissues over the thoracolumbar fascia, the second class the TLF itself, the third one the tissues below (including the epymisial fascia of the erector spinae muscles) (Fig. 2).

The images were first pre-processed. Specifically, the region of interest was cropped, 256 by 256 resized and normalized.

### Model specification

An established convolutional network architecture for ultrasound image segmentation, 2D U-Net [17, 19, 20, 24] was used to solve the segmentation task. This architecture has proven to be the most used in the segmentation of musculoskeletal structures [25].

### Model training/validation/testing

The proposed network was implemented using Keras framework and trained on Google Collaboratory. The net was trained on 451 images (as above-mentioned, divided proportionally into 80% training and 20% validation subsets), using categorical cross-entropy loss function with ADAM optimizer for 200 epochs, with batch size of 16.

### Model performance

On the testing subsets the segmentation performance was evaluated using: accuracy index, mean (averaged on the three classes) Intersection over Union coefficient (IoU) and Dice-score coefficient [25] Furthermore, two expert clinicians (F.G. and C.P.) for Test set 1 and one clinician (C.P.) for Test set 2 validated the segmentation results (network predictions) through visual inspection.

## Results

**Fig. 3:**
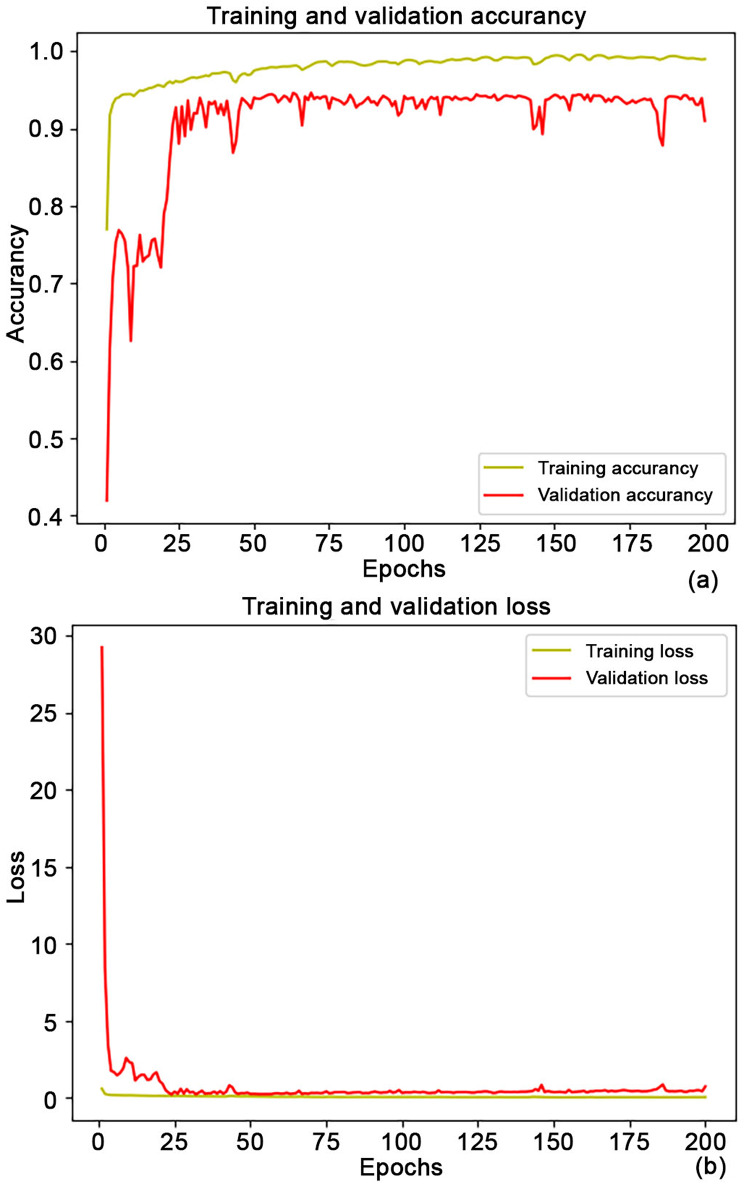
Training/Validation (**a**) accuracy and (**b**) loss from model

The segmentation achieved by the proposed model showed a final training/validation accuracy and loss of 0.99/0.91 and 0.02/0.71, respectively, as reported in Fig. 3.

The total time to train/validate the model was less than 120 min. The TLF segmentation network on a new test image could be performed approximately in real time (43 s to predict 87 images). Automated segmentation performance metrics yielded a mean IoU coefficient of 0.82 and a Dice-score of 0.76. The prediction accuracy on the test subset was 0.94. Meanwhile, the values obtained from the Test set 2 were a mean IoU index and Dice-score of 0.85 and 0.91, respectively.

**Fig. 4:**
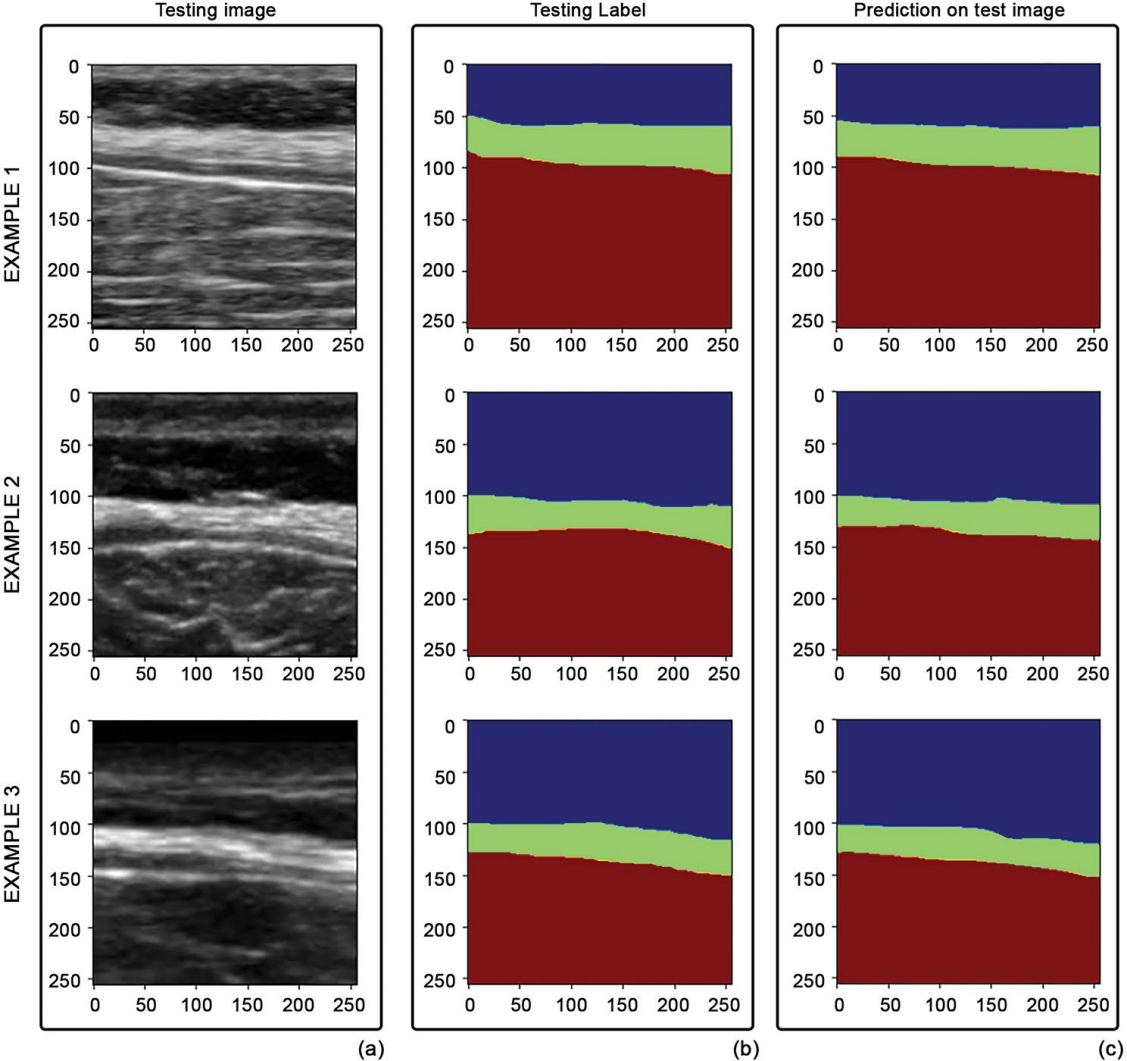
Examples of TLF segmentation: (**a**) original images, (**b**) clinicians’ annotations VS (**c**) model outputs

Furthermore, the results were verified by the two medical experts in the field, and both confirmed the validity of the model estimation through visual inspection. Some examples of TLF segmentation, from different testing images, are depicted in Fig. 4.

The trained network can segment the thoracolumbar fascia with all its layers. Moreover, the fascia can be distinguished from the surrounding structures, such as from the epimysial fascia of the erector spinae muscles.

## Discussion

In clinical practice, patients with LBP are studied through expensive clinical tests and imaging examinations (such as magnetic resonance imaging and computed tomography), which not always provide detailed information about the fascial tissues. On the other hand, ultrasound is gaining more and more interest as a fascia evaluation tool. Since it provides an objective tool to investigate myofascial tissue in real time, segmentation of ultrasound images could be extremely useful during clinical practice to identify and quantify alterations simply and quickly, resulting in cost reduction. To date, there is not an objective tool in literature that allows automatically investigating this structure in real time.

In literature, we could find example of ultrasound structures segmentation such as lower limb, upper limb, and so on [11–13, 15–20]. In this work, we segmented the thoracolumbar fascia from ultrasound images acquired from low-back-pain subjects, to respond to a clinical need that aims to support the investigation of this structure in an automatic and rapid way to evaluate its implications in conditions of musculoskeletal disease. Indeed, given the multifactorial nature of back pain and the need for a multidisciplinary approach [3], this work aims to contribute to the management of back pain by offering a new perspective for its automatic and rapid, real-time computational analysis during clinical routine (e.g., thickness assessment, targeted physical therapy on the segmented layer, changes after treatments, to name a few).

In term of implementation, it has been shown that computational choices resulting from using: data preprocessing steps such as normalization and resizing, dataset proportional splitting into subsets, the state-of-the-art U-Net model, a variant of the cross-entropy loss function, and the IoU/Dice-score as metrics, these are, in general, consolidated solutions to build up a tool for the segmentation of musculoskeletal structures from bioimaging [25].

In term of network, since the purpose of this study was to demonstrate the feasibility and potential of automatic TLF segmentation from low back subjects with a well-known state-of-the-art solution (i.e., U-Net), we did not focus on comparing the performance of the used algorithm with other state-of-the-art or recent artificial intelligence algorithms, however this comparison could be done as a future improvement.

In term of dataset, in this specific scenario no data augmentation was performed to avoid artificially increasing the sample size. Data augmentation operations such as affine transformation can be subject to unreliable representation of fascial structures. In fact, to demonstrate the generalizability of the method, an additional test set was introduced (Test set 2) that varied center, operator, machine manufacturer, patient cohort, and protocol. Despite the scarcity of images, the performance indicator results of Test set 2 (subjects without low-back pain) outperformed those of Test set 1 (subjects with LBP). This evidence opens the way for future studies (i.e., optimized network) aimed at understanding these structures in more depth. Further research into reliable data augmentation methods for fascial structures could be conducted in the future to mitigate the risk of overfitting and improve its robustness in real-world settings. Moreover, the dataset collection (Test set 1) was performed from a group of patients suffering from LBP, randomly selected during clinical practice and who showed high variability in anthropometric parameters and pain characteristics (i.e., pain level, disease duration and phenotype). This is a fundamental step, in fact, beyond the numerical values, the interpretation of the performance metrics should always be accompanied by an analysis of the initial dataset, since to be successful in clinical practice it should reflect the variability faced in the daily routine. Otherwise, the high-performance indicator values mislead its clinical utility and success in case of anatomical variant. Moreover, limitations in failure cases, especially in the case of complex images of lower quality (e.g., due to noise), are crucial for clinical application, since segmentation errors in critical areas can lead to misinterpretations. In the future, it will be possible to perform further simulations by adding a specific noise pattern to the test images. In term of validation, in the present study, the results showed promising metric indicators and clinical assessments from visual inspections. As a future work, quantitative evaluations or broader expert validation may be performed to increase reliability. For example, it is capable to identify the space between the TLF and the epymisial layer (identifying them as separate structures), and it is hypothesized that this interface is potentially one of the biomarkers of a proper layer gliding. One option to improve the robustness of the results could be to additionally expand the sample size by introducing additional variability (e.g., patient with specific pathological conditions) to assess this biomarker.

Furthermore, we hypothesize that since the training dataset used static images, if the algorithm is applied to video sequences, as in clinical routine, the error is potentially mitigated as the same structure is always analyzed in more than one frame.

The currently developed tool could offer an in-depth knowledge of fascia properties. Since fascia is acquiring a central role in the etiopathogenesis and treatment of musculoskeletal diseases (such as LBP), segmentation of the TLF could become an important task to understand its alterations and ultrasound patterns in patients with LBP. More specifically, an increase in fascial thickness has been associated with LBP and a reduction in fascial gliding between fascial surfaces resulted in patients with LBP compared to healthy subjects [1, 5, 21]. Therefore, additional post-processing steps may be performed in the future to quantify these properties.

Having precise data in terms of quantification of fascial thickness with its variations could highlight the importance of establishing practical precision protocols in bioimaging assessments. Fascia segmentation could be integrated in ultrasound devices for the evaluation both in research and in clinical practice to evaluate a specific region of interest before/during/after treatment and in follow-up, to direct medical intervention towards a treatment that is not only personalized but also site-specific.

## Conclusions

Segmentation of the thoracolumbar fascia using ultrasound imaging has shown promising outcomes, using a standard 2D U-Net architecture. Furthermore, thanks to the correct variability captured in the dataset, this tool will allow these results to be expanded into clinical practice. The findings of the proposed approach could pave the path for a successful confirmation of the fascia’s role in various nonspecific muscular pains that currently face challenges in being objectively identified.

## Data Availability

The datasets used and/or analysed during the current study are available from the corresponding author on reasonable request.

## Abbreviations

LBP: Low back pain
TLF: Thoracolumbar fascia
IoU: Intersection over union
